# Clinical and endoscopic characteristics of sessile serrated adenomas/polyps with dysplasia/adenocarcinoma in a Korean population: A Korean Association for the Study of Intestinal Diseases (KASID) multicenter study

**DOI:** 10.1038/s41598-019-40559-w

**Published:** 2019-03-08

**Authors:** Ki-Hyun Kim, Kyeong-Ok Kim, Yunho Jung, Jun Lee, Sang-Wook Kim, Jae-Hyun Kim, Tae-Jun Kim, Young-Seok Cho, Young-Eun Joo

**Affiliations:** 1 Department of Internal Medicine, Chonnam National University Medical School, Gwangju, Republic of Korea; 2 Department of Internal Medicine, Yeungnam University College of Medicine, Daegu, Republic of Korea; 3 Department of Internal Medicine, Soonchunhyang University College of Medicine, Cheonan, Republic of Korea; 4 Department of Internal Medicine, Chosun University College of Medicine, Gwangju, Republic of Korea; 5 Department of Internal Medicine, Chonbuk National University Medical School, Jeonju, Republic of Korea; 6 Department of Internal Medicine, Kosin University College of Medicine, Busan, Republic of Korea; 7 Department of Internal Medicine, Sungkyunkwan University College of Medicine, Seoul, Republic of Korea; 8 Department of Internal Medicine, Catholic University College of Medicine, Seoul, Republic of Korea

## Abstract

Sessile serrated adenomas/polyps (SSA/Ps) are precancerous lesions that account for one-third of colorectal cancers. The endoscopic and pathologic differentiation between SSA/Ps without dysplasia (SSA/POs) and SSA/Ps with dysplasia or adenocarcinoma (SSA/PDAs) can be difficult. This study aimed to assess the clinical characteristics of SSA/PDs. This multicenter retrospective cohort study included 532 patients who underwent endoscopic resection and were pathologically diagnosed with SSA/POs and SSA/PDAs. Initially, medical, endoscopic, and histopathological records of patients who underwent endoscopic resection of SSA/POs and SSA/PDAs at eight university hospitals in Korea between January 2005 and December 2015 were reviewed. A total of 307 (57.7%) patients were detected in men and 319 (60.0%) were located in the proximal colon. Most SSA/Ps had a flat, slightly elevated, or sessile morphology. The most prevalent endoscopic findings of SSA/Ps were nodular surface (244, 45.9%), disrupted vascular pattern (232, 43.6%), altered fold contour (141, 26.5%), dome-shaped morphology (135, 25.4%), and pale color (115, 21.6%). SSA/POs were more commonly found in the proximal colon, compared to SSA/PDAs. SSA/PDAs displayed 0-Ip, Isp, IIb or IIa + IIc morphologies more frequently, while SSA/POs displayed 0-Is or IIa morphology more frequently. The frequency of a rim of debris/bubbles was significantly higher in SSA/POs, while nodular surface and disrupted vascular pattern were significantly higher in SSA/PDAs. In the univariate analysis of endoscopic features, SSA/PDAs were significantly associated with the distal colon location, 0-Isp and IIb morphologies, nodular surface, and disrupted vascular pattern. In the multivariate analysis, 0-IIb, nodular surface, and disrupted vascular pattern were significantly associated with SSA/PDAs. SSA/Ps with 0-IIb morphology, nodular surface and disrupted vascular pattern are associated with an increased risk of dysplasia or adenocarcinoma.

## Introduction

Previously, two main groups of colorectal polyps were widely recognized: adenomatous polyps and non-adenomatous polyps, which include hyperplastic polyps (HPs). Conventional adenomatous polyps were traditionally considered the only precursor lesions of colorectal cancer, while non-adenomatous polyps were considered benign^1,2^. However, recent discoveries showed that colorectal serrated lesions with characteristic saw-tooth morphology of crypts, previously called HPs, lead to colorectal cancer via the serrated neoplasia pathway, which is different from the adenoma-carcinoma sequence pathway for conventional adenomatous polyps^3–7^. This pathway accounts for about 15–30% of sporadic colorectal cancers^3–7^. Molecular features of the serrated neoplasia pathway, including point mutations in the BRAF oncogene and methylation of CpG islands (CIMP) in the promoter regions of key regulatory and tumor suppressor genes, lead to epigenetic silencing of mismatch repair of genes such as *MLH1*, resulting in microsatellite instability (MSI)^3–7^.

Histopathologically, colorectal serrated lesions are classified into the following three general types according to the 2010 World Health Organization (WHO) classification: HPs, traditional serrated adenomas (TSAs), and sessile serrated adenomas/polyps (SSA/Ps)^8^. HPs are considered harmless with no malignant potential, while both TSAs and SSA/Ps are considered precursors of colorectal cancer^3–8^.

An interval colorectal cancer is defined as a cancer diagnosed prior to the date of the next recommended examination after a previous negative colonoscopy result. This cancer is thought to be resulted from previously missed lesions, rapid progression, or incomplete resection of colorectal precancerous lesions. Moreover, it is more likely to be associated with CIMP and MSI compared to non-interval colorectal cancer^9–11^.

SSA/Ps have more pale, flat, sessile, or indistinct borders compared to conventional adenomatous polyps. Therefore, they can be difficult to detect during colonoscopy and are commonly missed or incompletely resected^12–20^. Moreover, SSA/Ps are considered to have the potential of rapid progression owing to the development of MSI, especially after the development of cytological dysplasia^3–8^. These findings suggest that SSA/Ps are important contributors to interval colorectal cancers^9–11^. Therefore, SSA/Ps merit special attention to ensure optimal detection, complete resection, and appropriate surveillance. Previous studies have investigated the clinical and endoscopic features of SSA/Ps^12–20^; however, the clinical and endoscopic features of SSA/Ps with cytological dysplasia or adenocarcinoma have not yet been fully elucidated^21–30^.

This study aimed to evaluate the clinical and endoscopic characteristics of SSA/Ps and compare the characteristics of SSA/Ps without dysplasia with those of SSA/Ps with dysplasia or adenocarcinoma.

## Materials and Methods

### Study design and population

This retrospective, multicenter cohort study assessed consecutive patients with endoscopically resected and pathologically diagnosed lesions as either SSA/Ps without dysplasia (SSA/POs) or SSA/Ps with low-grade dysplasia, high-grade dysplasia, or adenocarcinoma (SSA/PDAs) at eight university hospitals throughout Korea affiliated with the Korean Association for the Study of Intestinal Disease between January 2005 and December 2015. One board-certified gastrointestinal endoscopist with extensive experience in endoscopic resections such as polypectomy, endoscopic mucosal resection (EMR), endoscopic piecemeal mucosal resection (EPMR), endoscopic submucosal dissection (ESD) at each hospital was responsible for data collection, and the completeness of the data collection was monitored by one of the authors (Y.E.J.). We excluded patients with lack of complete clinicopathological data and inflammatory bowel diseases, familial adenomatous polyposis, or non-epithelial neoplasms as carcinoid or lymphoma, and the presence of non-neoplastic histology such as chronic colitis. A total of 532 SSA/P lesions were retrospectively analyzed for various clinicopathological characteristics by reviewing medical, endoscopic, and histopathological records of enrolled patients. The patient-related factors including age, sex, smoking, alcohol drinking, smoking, body mass index (BMI), and use of aspirin and nonsteroidal anti-inflammatory drugs (NSAIDs), lesion-related factors including size, location, endoscopic morphology and feature, associated lesion, procedure-related factors including removal method and post-procedure complication and histologic factors were obtained by medical record reviews, pathologists, and gastroenterologists contact when necessary. Informed consent was obtained from every patients. The study was performed in accordance with the ethical principles of the Declaration of Helsinki and was approved by the Chonnam National University Hwasun hospital Institutional Review Board as well as by each Institutional Review Board at 7 hospitals (Yeungnam University Hospital Institutional Review Board, Soonchunhyang University Hospital Institutional Review Board, Chosun University Hospital Institutional Review Board, Chonbuk University Hospital Institutional Review Board, Kosin University Hospital Institutional Review Board, Samsung Medical Center Institutional Review Board, Seoul St. Mary’s Hospital Institutional Review Board).

### Endoscopic and histologic analysis of SSA/Ps

**Figure 1:**
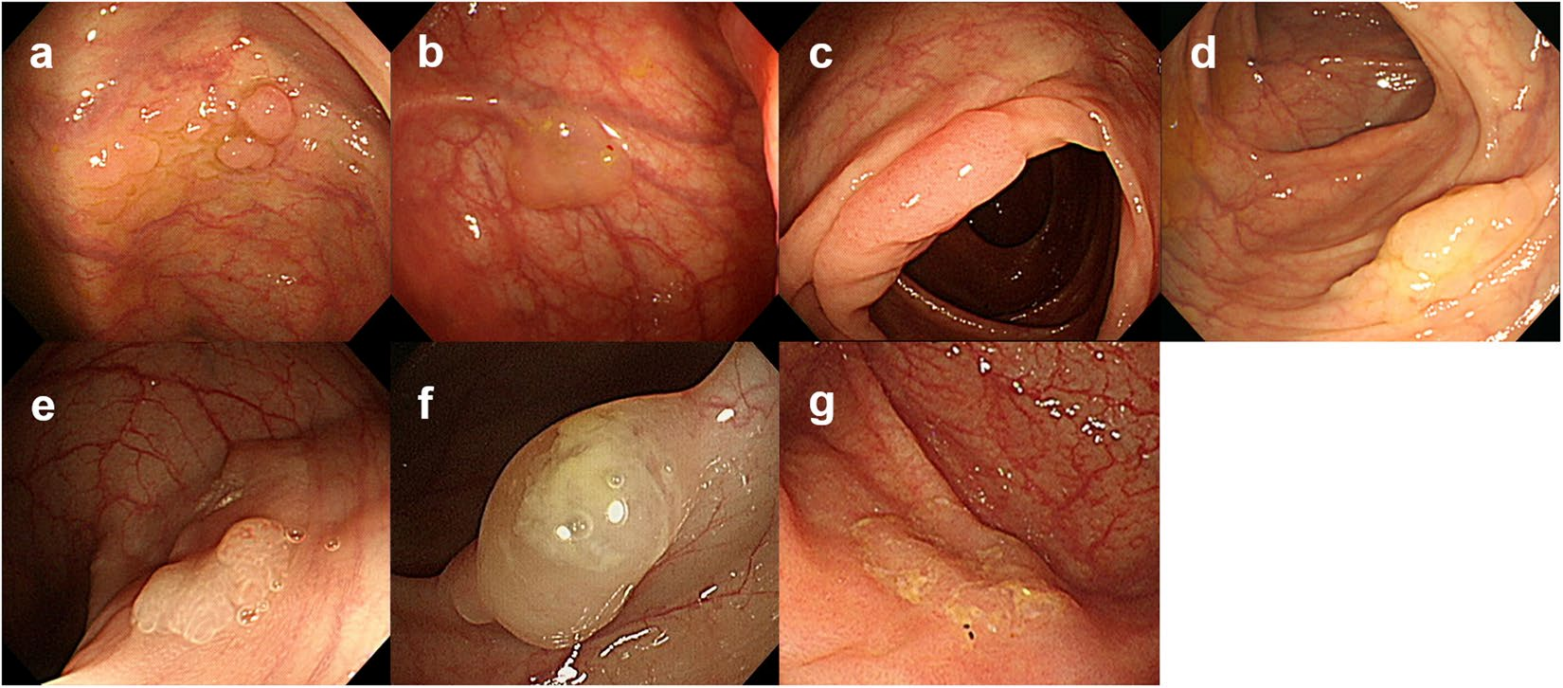
White-light colonoscopic images of sessile serrated adenomas/polyps (SSA/Ps) in a representative case. (**a**) Nodular surface. (**b**) Disrupted vascular pattern. (**c**) Altered fold contour. (**d**) Dome-shaped morphology. (**e**) Pale color. (**f**) Mucus cap. (**g**) Rim of debris/bubbles.

**Figure 2:**
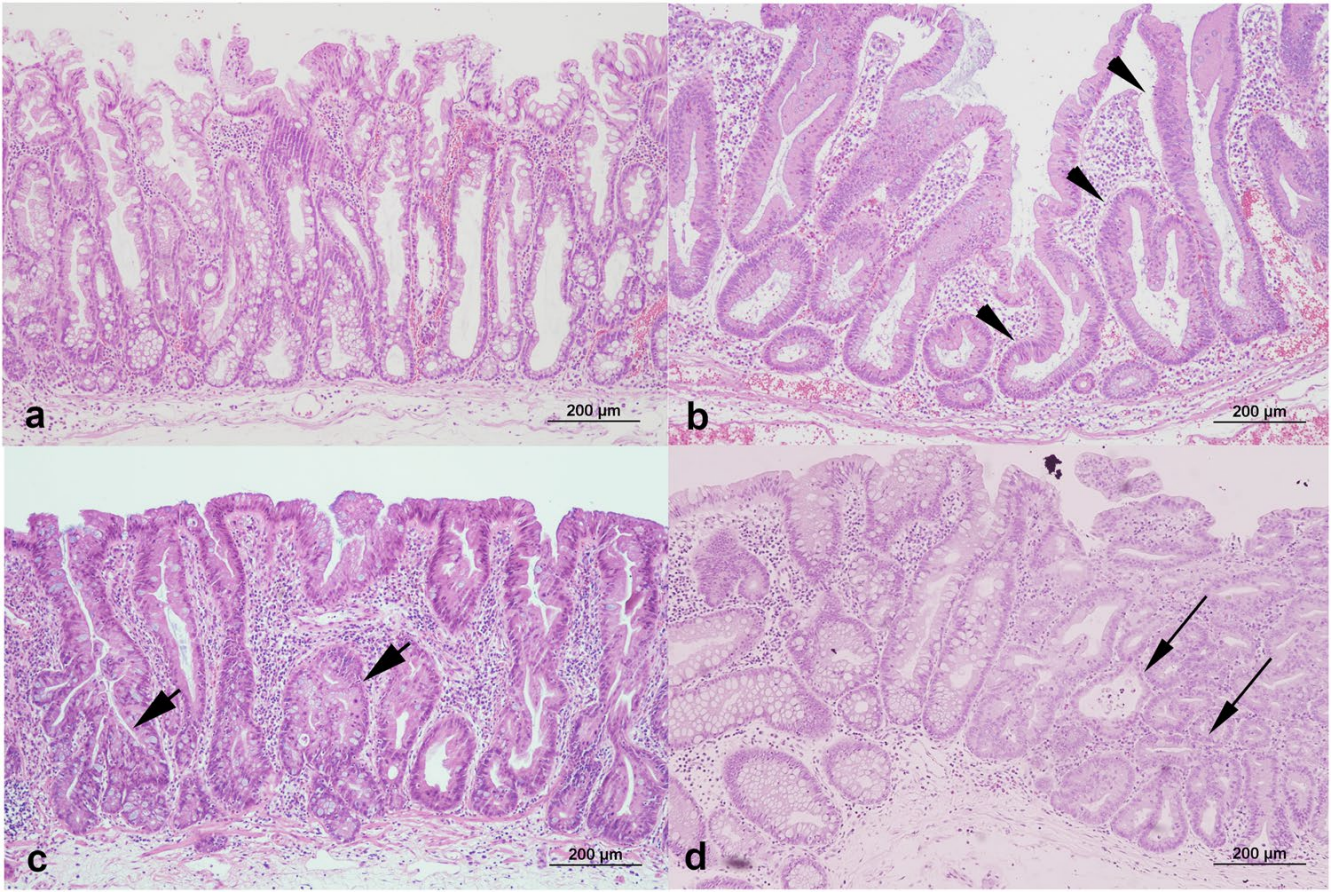
Histopatholgic findings with hematoxylin-eosin staining of the resected specimens of sessile serrated adenomas/polyps (SSA/Ps) (x100). (**a**) SSA/P without cytologic dysplasia shows the presence of serrated crypts, irregularly dilated and branching crypts, and horizontally and laterally arranged basal crypts. (**b**) SSA/P with low grade dysplasia (arrowhead). (**c**) SSA/P with high grade dysplasia (short arrow). (**d**) SSA/P with adenocarcinoma (long arrow).

All patients were examined using video colonoscopes (Olympus CF-240I or CF-H260; Olympus, Tokyo, Japan). Bowel preparation was performed with polyethylene glycol electrolyte solution in all hospitals and classified according to endoscopist estimation into adequate in all cases. Two endoscopists (K.H.K. and Y.E.J.) reviewed the endoscopic findings of SSA/Ps and evaluated conventional white-light colonoscopic images. Endoscopic characteristics were evaluated using previously validated criteria defined by Tadepalli *et al*.^31^: nodular surface, disrupted vascular pattern, altered fold contour, dome-shaped morphology, pale color, mucus cap, and rim of debris/bubbles (Fig. 1). According to the Paris classification, the endoscopic morphologies of superficial lesions are divided into three categories: protruding (0-I), non-protruding and non-excavated (0-II), and excavated (0-III). Type 0-I lesions are further subdivided into pedunculated (0-I), sessile (0-Is), or mixed (0-Isp); Type 0-II lesions are subdivided into slightly elevated (0-IIa), flat (0-IIb), or depressed (0-IIc)^32^. The locations of the adenomas were classified as follows: the proximal colon (cecum, ascending colon, hepatic flexure, and transverse colon) and distal colon (splenic flexure of the colon, descending colon, sigmoid, and rectum). Histologic diagnoses of SSA/POs and SSA/PDAs were evaluated separately by gastrointestinal pathologists who were blinded to the knowledge of original pathologic report at each institute on the basis of the 2010 WHO classification for the presence of serrated crypts, irregularly dilated and/or branching crypts, and horizontally and/or laterally arranged basal crypts^8^. SSA/Ps with cytological dysplastic changes were graded as low-grade dysplasia, high-grade dysplasia, or adenocarcinoma (Fig. 2). In case of any difference in histologic diagnosis, the two pathologists discussed the case until consensus was achieved.

### Statistical analysis

Clinical and endoscopic characteristics of SSA/POs and SSA/PDAs were compared using the chi-square test, Student’s *t*-test, or analysis of variance, as appropriate. Descriptive analyses included proportions for categorical data, as well as mean ± standard deviation (SD) for continuous data. Furthermore, a binary logistic regression model was used to identify the risk factors of SSA/PDs. All statistical analyses were performed with the Statistical Packages for the Social Sciences (SPSS, version 18.0; SPSS Inc., Chicago, IL, USA). A difference with *P* < 0.05 was considered statistically significant.

## Results

### Baseline characteristics of patients with SSA/P

The baseline characteristics of patients with SSA/P are summarized in Table 1. The mean age of enrolled patients was 58.0 ± 12.5 years (range, 23.0–90.0 years). The study group included 307 men (57.7%) and 225 women (42.3%). The mean SSA/P size was 10.8 ± 7.5 (range, 2.0–50.0) mm. Of the five hundred thirty two detected SSA/P lesions, three hundred nineteen lesions (60.0%) were localized in the proximal colon. According to Paris classification^32^, the numbers of subjects (percentage) in 0-Ip, 0-Isp, 0-Is, 0-IIa, 0-IIb, and 0-IIa + IIc classes were 52 (9.8%), 76 (14.3%), 234 (44.0%), 151 (28.4%), 5 (0.9%), and 14 (2.6%), respectively. No class 0-III lesion was detected. The frequencies of SSA/Ps endoscopic features were as follows: nodular surface (244, 45.9%), disrupted vascular pattern (232, 43.6%), altered fold contour (141, 26.5%), dome-shaped morphology (135, 25.4%), pale color (115, 21.6%), mucus cap (62, 11.7%), and rim of debris/bubbles (32, 6.0%). In the prevalence of synchronous colorectal neoplasms in patients with SSA/P, 210 (39.5%) patients had conventional adenoma with low-grade dysplasia, 129 (24.2%) had hyperplastic polyp, 31 (5.8%) had conventional adenoma with high-grade dysplasia, 30 (5.6%) had traditional serrated adenoma, and 24 (4.5%) had colorectal adenocarcinoma. SSA/Ps were resected by polypectomy such as cold biopsy or snare (79, 15.2%), EMR (412, 79.1%), EPMR (5, 1.0%), or ESD (20, 3.8%). The post-procedural bleeding rate was 6.3% (33/532) and the perforation rate was 0.4% (2/532). In histologic examination, 370 (69.6%) lesions were SSA/POs and 162 (30.4%) lesions were SSA/PDAs [139 low-grade dysplasia (26.1%), 16 were high-grade dysplasia (3.0%), and 7 were adenocarcinoma (1.3%)] (Table 1).

**Table 1:** Baseline characteristics of patients with sessile serrated adenoma/polyp.

Variables	n = 532 (%)
**Patient factors**	
Age (years) [mean ± SD] (range)	58.0 ± 12.5 (23.0–90.0)
Sex	
Male	307 (57.7)
Female	225 (42.3)
BMI (n = 499) [mean ± SD] (range)	24.1 ± 3.7 (14.4–55.0)
Alcohol (n = 498)	
No	297 (59.6)
Yes	201 (40.4)
Smoking (n = 500)	
No	336 (67.2)
Yes	164 (32.8)
Regular aspirin or NSAIDs use (n = 511)	
No	420 (82.2)
Yes	91 (17.8)
**Lesion factors**	
Size (mm) [mean ± SD] (range)	10.8 ± 7.5 (2.0–50.0)
Location	
Proximal colon	319 (60.0)
Distal colon	213 (40.0)
Location by subsites	
Cecum	53 (10.0)
Ascending colon	171 (32.1)
Transverse colon	95 (17.9)
Descending colon	38 (7.1)
Sigmoid colon	119 (22.4)
Rectum	56 (10.5)
Morphology (Paris classification)	
0-Ip	52 (9.8)
0-Isp	76 (14.3)
0-Is	234 (44.0)
0-IIa	151 (28.4)
0-IIb	5 (0.9)
0-IIa + IIc	14 (2.6)
Endoscopic features	
Nodular surface	244 (45.9)
Disrupted vascular pattern	232 (43.6)
Altered fold contour	141 (26.5)
Dome-shaped morphology	135 (25.4)
Pale color	115 (21.6)
Mucus cap	62 (11.7)
Rim of debris/bubbles	32 (6.0)
Associated lesions	
TSA	30 (5.6)
Hyperplastic polyp	129 (24.2)
Conventional adenoma with LGD	210 (39.5)
Conventional adenoma with HGD	31 (5.8)
ADC	24 (4.5)
**Procedure factors**	
Removal methods	
Polypectomy	79 (15.2)
EMR	412 (79.1)
EPMR	5 (1.0)
ESD	20 (3.8)
Post-procedural complications	
Bleeding	33 (6.3)
Perforation	2 (0.4)
**Histologic factors**	
SSA/POs	370 (69.6)
SSA/PDAs	162 (30.4)
SSA/P with LGD	139 (26.1)
SSA/P with HGD	16 (3.0)
SSA/P with ADC	7 (1.3)

SD, standard deviation; BMI, body mass index; NSAIDs, nonsteroidal anti-inflammatory drugs; TSA, traditional serrated adenoma; LGD, low-grade dysplasia; HGD, high-grade dysplasia; ADC, adenocarcinoma; EMR, endoscopic mucosal resection; EPMR, endoscopic piecemeal mucosal resection; ESD, endoscopic submucosal dissection; SSA/P, sessile serrated adenoma/polyp; SSA/POs, SSA/Ps without dysplasia; SSA/PDAs, SSA/Ps with dysplasia or adenocarcinoma.

### Comparison of clinical characteristics of SSA/POs and SSA/PDAs

SSA/Ps included 370 SSA/POs and 162 SSA/PDAs. With regard to patient-related factors, no statistically significant differences were found in age, sex, smoking, alcohol, or use of NSAIDs between SSA/POs and SSA/PDAs groups. The mean BMI value was significantly higher in SSA/POs than that of SSA/PDAs (P = 0.019). With regard to lesion-related factors, SSA/PDAs were more commonly found in the distal colon than in the proximal colon (P = 0.002). The distribution of SSA/POs and SSA/PDAs were also different according to location by subsites (P = 0.004). The proportion of rectosigmoid lesions among the subjects with SSA/PDAs (45%) was relatively higher than that among the subjects with SSA/POs (27.6%). No statistically significant differences were found in tumor size between SSA/POs and SSA/PDAs groups. The frequency of endoscopic morphology by Paris classification was different between SSA/POs and SSA/PDAs (P < 0.001). The analysis of endoscopic features showed that nodular surface and disrupted vascular pattern were more commonly found in SSA/PDAs (P < 0.001 and P = 0.006, respectively), and a rim of debris was more commonly found in SSA/POs (P = 0.023). Conventional adenomas with low-grade dysplasia were more commonly detected in SSA/PDAs than in SSA/POs (P < 0.001). Conventional adenomas with high-grade dysplasia and adenocarcinoma tended to be found more frequently in SSA/PDAs than in SSA/POs, but the difference was not statistically significant (P = 0.090). Traditional serrated adenomas were more commonly found in SSA/POs than in SSA/PDAs (P = 0.036). With regard to procedure-related factors, polypectomy, such as cold biopsy or snare, was more frequently performed in SSA/POs, while EMR was more frequently performed in SSA/PDAs (P = 0.001). Post-procedural bleeding was more commonly found in SSA/PDAs than in SSA/POs (P = 0.006) (Table 2).

**Table 2:** Comparison of clinical characteristics between sessile serrated adenomas/polyps without and with dysplasia or adenocarcinoma groups.

Variables	Frequency (%) or mean ± SD		P value
	SSA/POs (n = 370)	SSA/PDAs (n = 162)	
**Patient factors**			
Age (years) [mean ± SD] (range)	57.8 ± 12.4 (23.0–85.0)	58.6 ± 12.7 (31.0–90.0)	0.475
Sex			0.503
Male	210 (56.8)	97 (59.9)	
Female	160 (43.2)	65 (40.1)	
BMI (n = 499) [mean ± SD] (range)	24.3 ± 4.1 (14.3–55.0)	23.5 ± 3.2 (14.4–39.0)	0.019
Alcohol (n = 498)			0.629
No	202 (59.6)	95 (59.7)	
Yes	137 (40.4)	64 (40.3)	
Smoking (n = 500)			0.718
No	226 (66.3)	110 (69.2)	
Yes	115 (33.7)	49 (30.8)	
Regular aspirin or NSAIDs use (n = 511)			0.736
No	290 (83.1)	130 (80.2)	
Yes	59 (16.9)	32 (19.8)	
**Lesion factors**			
Location			0.002
Proximal	238 (64.3)	81 (50.0)	
Distal	132 (35.7)	81 (50.0)	
Location (Subsites)			0.004
Cecum	41 (11.1)	12 (7.4)	
Ascending colon	124 (33.5)	47 (29.0)	
Transverse colon	73 (19.7)	22 (13.6)	
Descending colon	30 (8.1)	8 (4.9)	
Sigmoid colon	72 (19.5)	47 (29.0)	
Rectum	30 (8.1)	26 (16.0)	
Size (mm) [mean ± SD] (range)	10.6 ± 7.4 (2.0–40.0)	10.9 ± 7.6 (2.0–50.0)	0.625
Proximal colon	11.4 ± 7.3	10.9 ± 7.6	0.350
Distal colon	9.2 ± 7.5	11.0 ± 7.6	0.825
Size (mm) [mean ± SD] (range) (Subsites)			
Cecum	10.6 ± 7.9	13.2 ± 10.0	0.717
Ascending colon	11.9 ± 6.7	10.4 ± 5.6	0.189
Transverse colon	11.0 ± 7.8	10.9 ± 9.7	0.872
Descending colon	9.4 ± 7.3	9.1 ± 4.8	0.486
Sigmoid colon	9.4 ± 7.8	10.7 ± 8.5	0.817
Rectum	8.6 ± 6.9	12.1 ± 6.8	0.615
Morphology (Paris classification)			<0.001
0-Ip	25 (6.8)	27 (16.7)	
0-Isp	47 (12.7)	29 (17.9)	
0-Is	169 (45.7)	65 (40.1)	
0-IIa	119 (32.2)	32 (19.8)	
0-IIb	2 (0.5)	3 (1.9)	
0-IIa + IIc	8 (2.2)	6 (3.7)	
Endoscopic features			
Nodular surface	127 (34.3)	117 (72.2)	<0.001
Disrupted vascular pattern	147 (39.7)	85 (52.5)	0.006
Altered fold contour	100 (27.0)	41 (25.3)	0.679
Dome-shaped morphology	98 (26.5)	37 (23.0)	0.394
Pale color	82 (23.2)	29 (17.9)	0.168
Mucus cap	46 (12.4)	16 (9.9)	0.398
Rim of debris/bubbles	28 (7.6)	4 (2.5)	0.023
Associated lesions			
TSA	26 (7.0)	4 (2.5)	0.036
Hyperplastic polyp	96 (25.9)	33 (20.4)	0.167
Conventional adenoma with LGD	126 (34.1)	84 (51.9)	<0.001
Conventional adenoma with HGD + ADC	26 (7.0)	19 (11.7)	0.090
Advanced adenoma	37 (10.0)	20 (12.3)	0.421
**Procedure factors**			
Removal method			0.001
Polypectomy	82 (22.2)	13 (8.0)	
EMR	271 (73.2)	141 (87.0)	
EPMR	4 (1.1)	1 (0.6)	
ESD	13 (3.5)	7 (4.3)	
Post-procedural complication			0.006
Bleeding	15 (4.1)	18 (11.2)	
Perforation	2 (0.5)	0 (0.0)	

SD, standard deviation; SSA/POs, SSA/Ps without dysplasia; SSA/PDAs, SSA/Ps with dysplasia or adenocarcinoma; BMI, body mass index; NSAIDs, nonsteroidal anti-inflammatory drugs; TSA, traditional serrated adenoma; LGD, low-grade dysplasia; HGD, high-grade dysplasia; ADC, adenocarcinoma; EMR, endoscopic mucosal resection; EPMR, endoscopic piecemeal mucosal resection; ESD, endoscopic submucosal dissection.

### Univariate analysis of risk factors associated with SSA/PDAs

The results of univariate analysis of risk factors associated with SSA/PDAs are summarized in Table 3. With regard to patient-related factors, no significant association with the risk of dysplasia was detected in terms of age, sex, smoking, alcohol drinking, BMI, and use of NSAIDs. With regard to lesion-related factors, SSA/PDAs were less commonly found in proximal colon, [odds ratio (OR) 0.555, 95% confidence interval (CI) 0.381–0.806, P = 0.002]. No significant association was found with tumor size. According to the Paris classification of endoscopic morphology, the risk of dysplasia was higher in 0-Isp (OR 2.295 95% CI 1.252–4.204, P = 0.007) and 0-IIb (OR 4.016, 95% CI 2.056–7.845, P < 0.001) morphologies compared to 0-IIa morphology. The analysis of endoscopic features showed that nodular surface and disrupted vascular pattern were positively associated with the risk of dysplasia (OR 4.975, 95% CI 3.317–7.461, P < 0.001; and OR 1.675, 95% CI 1.154–2.429, P = 0.007, respectively), while the rim of debris/bubbles was reversely associated with the risk of dysplasia (OR 0.309, 95% CI 0.107–0.897, P = 0.031). The risk of dysplasia was increased in subjects with conventional adenomas with low-grade dysplasia (OR 2.085, 95% CI 1.432–3.037, P < 0.001) and decreased in subjects with traditional serrated adenomas (OR 0.335, 95% CI 0.115–0.976, P = 0.045) (Table 3).

**Table 3:** Univariate logistic regression analysis of risk factors associated with sessile serrated adenomas/polyps with dysplasia or adenocarcinoma.

Variables	Univariate analysis		
	Odds ratio	95% CI	P value
**Patient factors**			
Age (years)	1.005	0.991–1.020	0.474
Sex			
Male *vs*. Female	1.137	0.781–1.655	0.503
BMI	0.938	0.889–0.990	0.020
Alcohol			
No	Ref		
Yes	0.993	0.677–1.458	0.973
Smoking			
No	Ref		
Yes	0.875	0.584–1.312	0.519
Regular aspirin or NSAIDs use			
No	Ref		
Yes	1.210	0.751–1.950	0.434
**Lesion factors**			
Location			
Proximal colon	0.555	0.381–0.806	0.002
Distal colon	Ref		
Size	1.006	0.982–1.031	0.625
Morphology (Paris classification)			
0-Ip	5.578	0.894–34.818	0.066
0-Isp	2.295	1.252–4.204	0.007
0-Is	1.430	0.882–2.320	0.147
0-IIa	Ref		
0-IIb	4.016	2.056–7.845	<0.001
0-IIa + IIc	2.789	0.903–8.618	0.075
Endoscopic features (Yes *vs*. No)			
Nodular surface	4.975	3.317–7.461	<0.001
Disrupted vascular pattern	1.675	1.154–2.429	0.007
Altered fold contour	0.915	0.600–1.395	0.679
Dome-shaped morphology	0.828	0.537–1.278	0.394
Pale color	0.720	0.451–1.150	0.169
Mucus cap	0.772	0.423–1.409	0.399
Rim of debris/bubbles	0.309	0.107–0.897	0.031
Associated lesions (Yes *vs*. No)			
TSA	0.335	0.115–0.976	0.045
Hyperplastic polyp	0.730	0.467–1.142	0.168
Conventional adenoma with LGD	2.085	1.432–3.037	<0.001
Conventional adenoma with HGD + ADC	0.569	0.305–1.060	0.076
Advanced adenoma	1.268	0.711–2.260	0.422
**Procedure factors**			
Post-procedural complication			
Bleeding (Yes *vs*. No)	2.912	1.428–5.937	0.003

CI, confidence interval; BMI, body mass index; NSAIDs, nonsteroidal anti-inflammatory drugs; TSA, traditional serrated adenoma; LGD, low-grade dysplasia; HGD, high-grade dysplasia; ADC, adenocarcinoma.

### Multivariate analysis of risk factors associated with SSA/PDAs

The results of the multivariate analysis of risk factors associated with SSA/PDAs are summarized in Table 4. On multivariate logistic regression analysis, SSA/Ps with 0-IIb morphology, nodular surface, or disrupted vascular pattern showed a significant association with the risk of dysplasia (OR 3.107, 95% CI 1.447–6.671, P = 0.004; OR 4.686, 95% CI 2.962–7.414, P < 0.001; and OR 1.770, 95% CI 1.150–2.724, P = 0.009, respectively) (Table 4).

**Table 4:** Multivariate logistic regression analysis of risk factors associated with sessile serrated adenomas/polyps with dysplasia or adenocarcinoma.

Variables	Multivariate analysis		P value
	Odds ratio	95% CI	
BMI	0.947	0.893–1.004	0.070
Location			
Proximal colon	0.664	0.426–1.037	0.072
Distal colon	Ref		
Morphology (Paris classification)			
0-Ip	6.758	0.891–51.273	0.065
0-Isp	1.840	0.918–3.690	0.086
0-Is	1.579	0.912–2.733	0.103
0-IIa	Ref		
0-IIb	3.107	1.447–6.671	0.004
0-IIa + IIc	1.365	0.407–4.581	0.614
Endoscopic features			
Nodular surface	4.686	2.962–7.414	<0.001
Disrupted vascular pattern	1.770	1.150–2.724	0.009
Altered fold contour	1.280	0.786–2.084	0.322
Dome-shaped morphology	1.069	0.636–1.795	0.802
Pale color	0.845	0.487–1.465	0.548
Mucus cap	1.070	0.535–2.140	0.849
Rim of debris/bubbles	0.462	0.144–1.486	0.195

CI, confidence interval; BMI, body mass index.

### Comparison of tumor size between SSA/POs and SSA/PDAs groups according to removal method and age groups

The results of the comparison of tumor size between SSA/POs and SSA/PDAs groups according to removal methods are summarized in Table 5. The tumor size of SSA/Ps treated by polypectomy, such as cold biopsy or snare, was 6.2 ± 5.9 mm, by EMR, 10.6 ± 6.0 mm, by EPMR, 27.0 ± 8.3 mm, and by ESD, 27.5 ± 12.6 mm. The comparison of tumor size between SSA/POs and SSA/PDAs groups according to removal method and age groups showed statistically non-significant differences among applied removal methods (Table 5).

**Table 5:** Comparison of tumor size between sessile serrated adenomas/polyps without and with dysplasia or adenocarcinoma groups according to removal method and age groups.

Removal methods	Tumor size of SSA/Ps (mm) [mean ± SD] (range)		
**Total (n = 532)**			
Polypectomy (n = 95)	6.2 ± 5.9 (2.0–30.0)		
EMR (n = 412)	10.8 ± 6.0 (2.0–40.0)		
EPMR (n = 5)	27.0 ± 8.3 (15.0–35.0)		
ESD (n = 20)	27.5 ± 12.6 (6.0–50.0)		
**Variables**	**SSA/POs (n = 370)**	**SSA/PDAs (n = 162)**	**p-value**
**Tumor size** (n) (%)			
<10 mm (n = 271)	194 (52.4)	77 (47.5)	0.268
10–20 mm (n = 190)	124 (33.5)	66 (40.7)	0.271
≥20 mm (n = 71)	52 (14.1)	19 (11.7)	0.700
**Age [mean ± SD]** (n)			
Age <58 (n = 263)			
Polypectomy (n = 46)	4.5 ± 2.7 (39)	7.0 ± 4.5 (7)	0.210
EMR (n = 209)	10.9 ± 5.8 (142)	10.1 ± 5.5 (67)	0.337
EPMR (n = 2)	27.0 ± 7.0 (2)	0(0)	NA
ESD (n = 6)	32.5 ± 8.6 (4)	46.0 ± 5.6 (2)	0.100
Age ≥58 (n = 269)			
Polypectomy (n = 49)	6.8 ± 6.6 (43)	11.6 ± 8.1 (6)	0.213
EMR (n = 203)	11.2 ± 6.4 (129)	10.2 ± 5.7 (74)	0.257
EPMR (n = 3)	33.0 ± 2.8 (2)	15.0 (1)	0.121
ESD (n = 14)	23.5 ± 9.1 (9)	23.2 ± 16.5 (5)	0.966

SD, standard deviation; SSA/Ps, sessile serrated adenoma/polyps; EMR, endoscopic mucosal resection; EPMR, endoscopic piecemeal mucosal resection; ESD, endoscopic submucosal dissection; SSA/POs, SSA/Ps without dysplasia; SSA/PDAs, SSA/Ps with dysplasia or adenocarcinoma; NA, not available.

## Discussion

SSA/Ps are considered major precursor lesions of the serrated neoplasia pathway, which account for one-third of all sporadic colorectal cancers^3–8^. SSA/Ps comprise approximately 15–25% of colorectal serrated lesions and 2–9% of all colorectal polyps. SSA/Ps have a marked predilection for the proximal colon and have predominantly a sessile or flat morphology^12–20^. In our study, 60.0% of SSA/Ps were localized in the proximal colon and 75.9% had sessile (0-Is) or flat morphologies (0-IIa, 0-IIb, and 0-IIa + IIc). These results are similar to those revealed by previous^12–20^ and our studies. Histopathologically, SSA/Ps are subclassified as SSA/POs and SSA/PDAs, and SSA/PDAs accounted for about 15.1% of SSA/Ps and 0.18% of all colorectal polyps in a large cohort study^33^. In our study, the incidence of SSA/PDs was 30.4% of SSA/Ps. This result is inconsistent with that of another report^33^ and may be related to the variable sample size of the current study and the previous report and the unavoidable selection bias of the present retrospective study.

Endoscopic detection of SSA/Ps is difficult because of their subtle features compared to conventional adenomas^12–20^. Previously, the most prevalent endoscopic features of SSA/Ps according to the criteria defined by Tadefalli *et al*. were mucous cap, rim of debris/bubbles, altered fold contour, and disrupted vascular pattern^31^. Another study showed that the disrupted vascular pattern, altered fold contour, or rim of debris/bubbles were most prevalent endoscopic features of SSA/Ps^21^. In our study, the main endoscopic features of SSA/Ps were nodular surface, disrupted vascular pattern, or altered fold contour.

Before the development of cancer, SSA/Ps progress indolently; however, they are believed to progress rapidly to cancer after the beginning of cytological dysplasia^3–8^. Therefore, the differentiation between SSA/POs and SSA/PDAs is clinically important. Especially, accurate endoscopic differentiation is very important and can eventually result in complete resection via immediate decision-making of the resection of target lesions. Moreover, the complete *en bloc* resection may be basically indicated for SSA/PDAs, regardless of tumor size^12–20^.

SSA/PDAs occur frequently in older female patients and in the proximal colon^21–30^. In our study, no significant differences were detected between SSA/POs and SSA/PDAs in terms of age and sex, but SSA/PDAs affected predominantly the distal colon.

SSA/PDAs are clinically and endoscopically similar to SSA/POs, making endoscopic differentiation difficult. A previous study showed that endoscopic features of SSA/POs tended to present as more often altered fold contour and SSA/PDAs tended to be more often characterized by a pale color and dome-shaped morphology than SSA/POs^21^. Another study reported that the incidence of any 0-Is morphologies or nodular components within the lesions was higher for SSA/PDAs than for SSA/POs^29^. Moreover, in SSA/PDAs with nodule/protrusion, the nodule/protrusion detected by endoscopy corresponded to the portion of dysplasia or carcinoma on histology^28^. Furthermore, SSA/PDAs displayed pedunculated (0-Ip) or semipedunculated (0-Isp) morphologies more frequently than SSA/POs^30^. In our study, nodular surface, disrupted vascular pattern, and 0-Isp or 0-IIb morphologies were more commonly found in SSA/PDAs, while rim of debris/bubbles was more commonly found in SSA/POs in analysis of endoscopic features. On multivariate analysis, SSA/Ps with 0-IIb, nodular surface, or disrupted vascular pattern showed the significant association with the risk of dysplasia or adenocarcinoma. The analyses of sensitivity and specificity of each characteristic for SSA/PDs showed the results as follow; SSA/Ps with 0-IIb (sensitivity 1.9% and specificity 99.5%), nodular surface (sensitivity 72.2% and specificity 65.7%), and disruption of vascular pattern (sensitivity 52.5% and specificity 60.3%). Nodular surface showed a better predictive performance than others. Therefore, because SSA/Ps with these findings are likely to be accompanied by dysplasia or adenocarcinoma, they should be removed *en bloc* for accurate histopathological assessment and prevention of interval cancer development.

The presence of SSA/Ps was associated with the presence of synchronous advanced colorectal neoplasia^34–36^. In our study, 49.8% of patients with SSA/Ps had conventional adenoma with low-grade dysplasia, high-grade dysplasia, or adenocarcinoma. Furthermore, conventional adenomas with low-grade dysplasia were more commonly found in SSA/PDAs than in SSA/POs. Advanced adenomas with high-grade dysplasia and adenocarcinoma tended to be found more frequently in SSA/PDAs compared to SSA/POs. Therefore, the recognition of SSA/Ps should alert the endoscopist to meticulously inspect the remaining part of the colonic mucosa for more lesions.

In our study, SSA/Ps were resected by various techniques, including polypectomy, such as cold biopsy or snare, EMR, EPMR, or ESD as endoscopic resection of conventional adenoma^37–40^. Moreover, treatment was applied according to the tumor size, namely polypectomy was usually used for resection of SSA/Ps < 10 mm, EMR was used for SSA/Ps 10–20 mm, and ESD or EPMR was used for SSA/Ps ≥ 20 mm. In addition, the comparison of tumor size between SSA/POs and SSA/PDAs groups according to removal method and age groups showed no statistically significant differences between applied removal methods. Although the treatment strategy for SSA/Ps has not yet been established, our results showed that the principles for the management of SSA/Ps may be similar to those for conventional adenomas in clinical practice. Post-procedural bleeding was more commonly found in SSA/PDAs than in SSA/POs. This may be related to the fact that the lesions with dysplasia or adenocarcinoma present rapid growth and increased neovascularization toward the submucosa.

However, our study has some limitations. First, the study design was retrospective and nonrandomized; therefore, selection biases were unavoidable. Second, the heterogeneity of the SSA/PO and SSA/PDA groups was inevitable. For these reasons, large prospective, multicenter studies evaluating the clinical and endoscopic characteristics and outcomes of SSA/PDAs for optimal detection, complete resection, and appropriate surveillance of SSA/Ps are needed to provide more definitive evidence.

In conclusion, SSA/Ps with 0-IIb, nodular surface and disrupted vascular pattern are associated with an increased risk of dysplasia or adenocarcinoma. Therefore, these findings can be considered useful indicators in the management of SSA/Ps.
